# A Unique Message

**Published:** 1887-06

**Authors:** 


A UNIQUE MESSAGE.—The following message was written, a few days ago, on the slate of a well known phys'cian of this city :

"*Take Warning!* To all to whom it may concern : Anybody's oxes or cows found in this here patch, his or her's tails will be cut off as the case may be!"

The Doctor thinks it was written by an ungrateful cow-boy, lately returned from Texas, upon whom, a short while ago, he performed the operation of circumcision. E. v. G.

